# Host spatiotemporal overlap in a park with high endemicity of *Echinococcus multilocularis*

**DOI:** 10.3389/fpara.2023.1161108

**Published:** 2023-03-13

**Authors:** Darcy R. Visscher, Emilie Toews, Jesse Pattison, Philip D. Walker, Colborne Kemna, Marco Musiani, Alessandro Massolo

**Affiliations:** ^1^ Department of Biology, The King’s University, Edmonton, AB, Canada; ^2^ Department of Biological Science, University of AB, Edmonton, AB, Canada; ^3^ Naturalis Biodiversity Center, Leiden, Netherlands; ^4^ Department of Ecosystem and Public Health, Faculty of Veterinary Medicine, University of Calgary, Calgary, AB, Canada; ^5^ Department of Biology, University of Pisa, Pisa, Italy; ^6^ UMR CNRS 6249 Chrono-environnement, Université Bourgogne Franche-Comté, Besançon, France

**Keywords:** coyotes, domestic dog, *Echinococcus multilocularis*, remote camera, transmission risk, zoonotic parasites

## Abstract

**Background:**

There has been a spate of recent cases of human alveolar echinococcosis (AE) in Alberta, Canada. Alveolar echinococcosis is caused by *Echinococcus multilocularis*, which is prevalent among coyote populations and present in domestic dogs in Alberta.

**Methods and results:**

Using qPCR, we estimated the seasonal fecal prevalence of *E. multilocularis* in coyotes and dogs in a multiuse recreation area close to Edmonton, Alberta, where we also setup remote cameras to model seasonal changes in the overlap in temporal activity and the spatial intensity of use among coyotes, humans, and dogs, as a proxy of potential transmission. We detected *E. multilocularis* in 18 of 137 wild canid feces and none in 44 dog feces. After correcting for the qPCR test’s sensitivity and specificity, we estimated at 15.7% (9.7-22.7%, 95% CrI) the true fecal prevalence for coyotes. Temporal overlap between coyotes and both humans and dogs increased in the fall and winter relative to the spring and summer. Coyote intensity of use showed seasonal variations and was higher on maintained trails and locations closer to visitor parking and at sites with high intensity of dog use.

**Conclusions:**

Our results reinforce the need of an integrated approach, typical of both One-Health and Eco-Health, to park management for minimizing the likelihood of transmission where human and dog activity results in significant overlap with the one of the natural definitive hosts of zoonotic parasites.

## 1 Introduction

Continued urbanization and land use change has the potential to facilitate the transmission of zoonoses to humans through increased contact with synanthropic wildlife, either directly or indirectly *via* domestic pets (Bradley & Altizer, 2007; Jones et al., 2008; Hassell et al., 2017; Plowright et al., 2021). In response to this increased potential for zoonotic transmission there has been a growing understanding that holistic approaches encompassing veterinary medicine and ecology are required at the human–animal–environment interface to protect public health (Webster et al., 2016; Lerner and Berg, 2017; Plowright et al., 2021). These paradigms, including One-health and Eco-health, seek to understand how human behaviour, host ecology, and landscapes promote potential routes of zoonotic transmission and modify the risks to public health, particularly for emerging zoonoses (Webster et al., 2016; Lerner and Berg, 2017; Plowright et al., 2021).

*Echinococcus multilocularis* is a tapeworm of emerging concern and is the etiological agent for alveolar echinococcosis (AE hereafter) in humans. While AE was once rare in North America, since 2013 at least 17 human cases have been confirmed by hospitals in Alberta, Canada (Massolo et al., 2014; Massolo et al., 2019; Houston et al., 2021). The *E. multilocularis* lifecycle includes small rodents as intermediate hosts and wild or domestic canids (e.g., foxes, coyotes, dogs), which prey upon these mammals, as definitive hosts. In urban settings, wild canids may indirectly and incidentally interact with humans and domestic dogs, potentially facilitating the transmission of these parasites (Eckert et al., 2001; Deplazes et al., 2004; Bradley & Altizer, 2007; Umhang et al., 2014; Liccioli et al., 2015; Romig et al., 2017). Human infection may result from the accidental ingestion of eggs normally excreted in infected canid faeces, either directly or through contaminated soil (Torgerson et al., 2020).

Although dogs with *E. multilocularis* infection have a lower worm burden than wild canid hosts, their close association with humans may increase the risk of transmission (Kapel et al., 2006, Torgenson and Heath, 2003, Torgerson et al., 2020; Houston et al., 2021; Toews et al., 2021). Indeed, dog ownership in endemic areas is considered as a risk factor in the development of human AE (Kern et al., 2004; Conraths et al., 2017; Toews et al., 2021). Therefore, domestic dogs may play an important role in linking the sylvatic and synanthropic lifecycles of *E. multilocularis*, and this link may be influenced by the recreational activities of the owners where those activities are conducted, and the behavioural response of wildlife (Deplazes et al., 2004; Thompson, 2013; Hegglin et al., 2015; Liccioli et al., 2015). Consequently, it is crucial to examine the ecological and behavioural factors that may facilitate transmission of *E. multilocularis* to domestic dogs (Torgerson et al., 2020; Houston et al., 2021; Polish et al., 2021). If recreational activities have the potential to increase zoonotic contact, it is essential that we assess both the prevalence of *E. multilocularis* in frequented multiuse recreational areas, and the spatiotemporal activity of definitive and accidental hosts to determine the potential role they play in transmission (Deplazes et al., 2004; Mori, 2020).

In this paper we aimed to contemporarily estimate the prevalence of the zoonotic parasite *E. multilocularis* in wild canids and dogs walked in the Cooking Lake-Blackfoot Provincial Recreation Area (hereafter BPRA) and model the seasonal and spatial patterns of intensity of use and overlap in activity in this multiuse park by coyotes, domestic dogs, and humans, and discuss its implications for the potential transmission and management of *E. multilocularis*.

## 2 Methods

### 2.1 Study area

The Cooking Lake-Blackfoot Provincial Recreation Area (BPRA) is a 90km^2^ multiuse natural park located approximately 40km east of Edmonton, capital of Alberta, Canada and its 1-million inhabitants. It is found within the Beaver Hills Biosphere Reserve, which was designated as such by the United Nations Educational, Scientific, and Cultural Organization (UNESCO; https://en.unesco.org/biosphere/eu-na/beaver-hills) and is predominately comprised of aspen parkland forest interspersed with small patches of coniferous forest. BPRA staff maintain trails (in both summer and winter) throughout the BPRA for nonmotorized use (hiking, biking, cross-country skiing, equestrian use, and hunting in the fall). Access and parking for the BPRA is located at four staging areas with visitor parking, from which most of the trails originate. Approximately half of the BPRA has been converted to fenced pastures for seasonal cattle grazing.

### 2.2 Camera trapping

**Figure 1:**
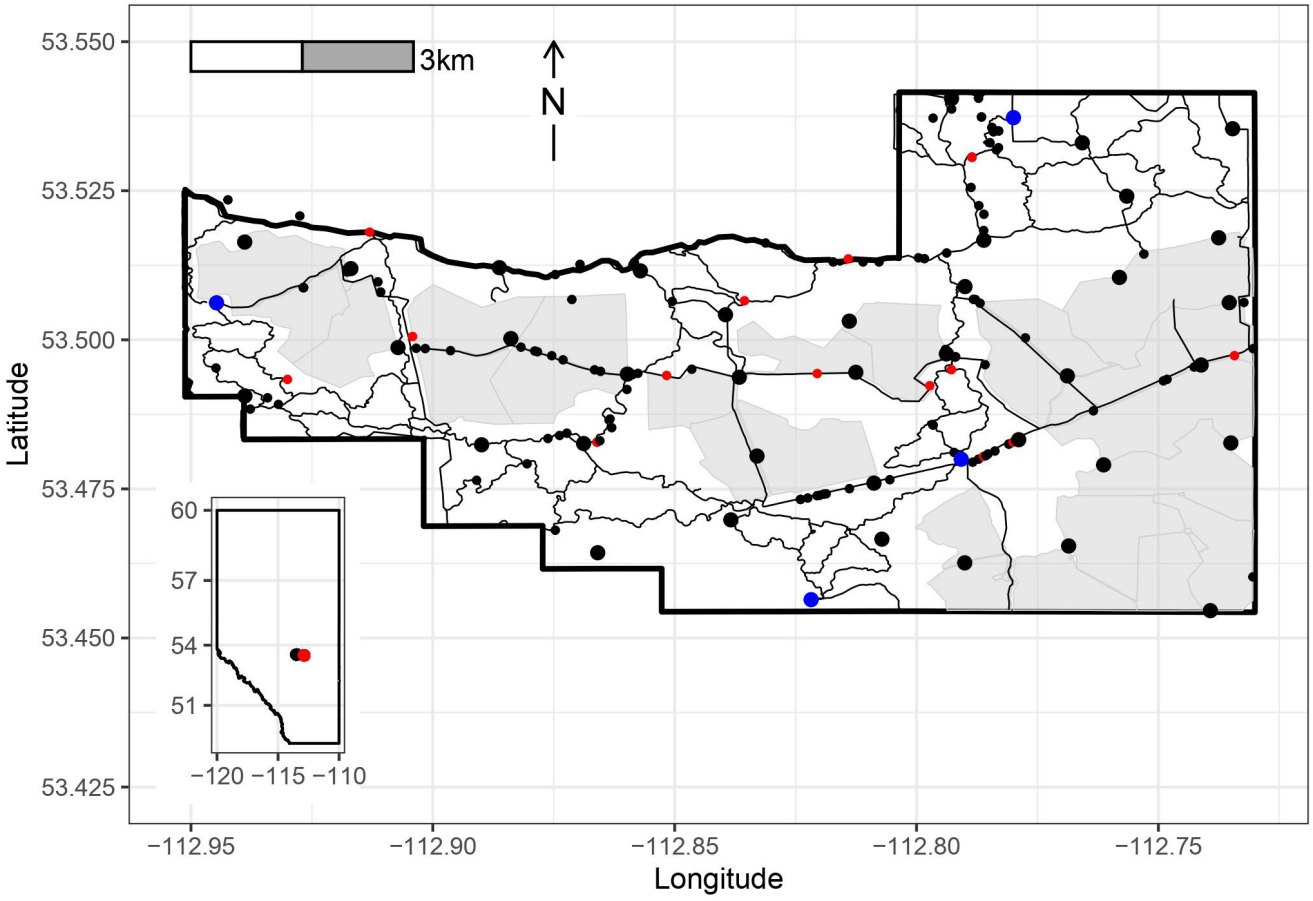
Blackfoot-Cooking Lake Provincial Recreation Area (BPRA, boundary in thick black line) Alberta, Canada, with the locations of 37 remote cameras indicated by large black dots, visitor parking in large blue dots, the predominantly open and fenced fields in light grey, and maintained trails and roads in thin black lines. Coyote fecal samples are given in the small black dots and positive samples are indicated in red. Inset map shows the study area (red dot) relative to the province of Alberta and the city of Edmonton (black dot).

We deployed 37 camera traps (Reconyx Hyperfire: H500, P800, P900) randomly within a 1600m size grid, with locations being constrained to be no closer than 800m from the nearest next camera and accessible for regular checking (**Figure 1**). Each camera was attached to a tree approximately 1m off the ground and were placed facing areas where detections would be maximized, such as trails or open areas. Cameras were set to take three photos with each motion triggered event and were serviced every 2 months with batteries being refreshed as needed, and the SD cards replaced. We collected camera trap data from June 2017 to July 2018. The EventFinder suite was used to facilitate the removal of non-target (i.e. vegetation and empty frames) images and then used to collapse individual images into independent events for classification (for full details see Janzen et al., 2019). Photo metadata including camera name, location, date, and temperature were recorded and the events were tagged with species name, age class, sex, and number of individuals.

### 2.3 Biological sample collection

We collected wild canid scat opportunistically across each season as we traversed the park performing camera maintenance. Scats were only collected if deemed to be fresh based on our trail revisitation time or scat characteristics. All scats used in the analysis were deemed to be less than a week old. Scats were collected in labelled zip top bags and the GPS coordinates recorded. At the end of the field day scats were transferred to a -18°C freezer. Following collection, all samples were frozen at -80°C for 96 hours in order to kill any virulent *Echinococcus* eggs that could be present in the samples (Veit et al., 1995). Samples were subsequently stored at -18°C until they were analysed. Scat dimensions is a relatively poor metric to determine if a scat is a wolf or coyote (or fox), so we refer to all scats as having come from wild canids. However, we note that the camera traps only infrequently recorded at most a pair of wolves (46 wolf events compared to 3459 coyote events), and only one image of a fox was recorded, thus the results from our wild canid scats are representative of coyotes.

From May to September 2018, we obtained fecal samples from dogs being walked in the BPRA and had dog owners fill out a survey. We used a modified version of the dog owner survey employed by Smith et al. (2014) which was divided into several sections (see the **supplemental data**; Smith et al., 2014; Porter et al., 2022). The first section contained screening questions for inclusion in the survey, which included owning the dog in question, being a repeat user of the BPRA, the owner being over the age of 18, and the dog being over 6 months in age. The second section focused on dog demographic details including gender, breed, age class, spay/neuter status, as well as veterinary care and deworming practices. The following sections were specific to dog owner behavior, which included questions about dog walking routines, levels of off leash activity, and the prey drive and scavenging activity of the dog. We used a Likert type ranked 6-point scale to record the frequency of walking behavior at different types of location and the frequency of off-leash activity at those locations. To be included in the study, a completed survey and fecal sample needed to be provided. Participants were asked to collect a fresh fecal sample and leave it with the researcher after completing their walk. Fecal samples were stored in coolers until the end of the day then frozen at -18°C. Following the summer collection period, all samples were frozen at -80°C for 96 hours in order to kill any virulent *Echinococcus* eggs that could be present in the samples (Veit et al., 1995). Samples were then stored at -18°C until they were analysed.

All procedures performed in studies involving human participants were in accordance with the ethical standards of The King’s University research ethics board. Informed consent was obtained from all individual participants included in the study. All applicable institutional and/or national guidelines for the non-invasive use of animals were followed.

### 2.4 Data analysis

#### 2.4.1 *Echinococcus multilocularis* presence and prevalence estimate

We extracted DNA from 200mg of each fecal sample using the Omega Mag-Bind^®^ Universal Pathogen DNA extraction kit (#M4029-01) following the manufacturer’s instructions, as well as the addition of five cycles of freezing with dry ice for 1 minute and heated at 70°C for 1 minute between the initial homogenization step and the addition of proteinase K to the sample to aid in the release of DNA from the shell of *E. multilocularis* egg. We then used automated DNA extraction and determined *E. multilocularis* presence using a duplex qPCR reaction of Nad243 primers (Santa et al., 2019) to amplify the mitochondrial gene *nad2* and an internal amplification control (Deer et al., 2010) to determine the presence of PCR inhibitors in the sample.

To estimate the true prevalence of *E. multilocularis*, we used a Bayesian approach (Speybroeck et al., 2013; Flor et al., 2020; Toews et al., 2021) using the R package ‘*prevalence’* (Devleesschauwer et al., 2014; R Core Team, 2021). We estimated the true seasonal prevalence based on published specificity and sensitivity values of the qPCR test we employed, 100% and 87.1%, respectively, using uniform priors for both specificity (90.0-99.9) and sensitivity (70.2-96.4; Santa et al., 2019; Toews et al., 2021). In all cases the prevalence model was implemented using two chains containing 10,000 “burn-in” samples and 10,000 samples that were retained, a multivariate Brooks-Gelman-Rubin statistic was inspected to ensure model convergence. For true prevalence estimates we report the 2.5% and 97.5% credibility intervals; for situations with 0 positive cases we report the 0% and 95% credibility intervals (following Toews et al., 2021 and Porter et al., 2022). We subsequently tested for seasonal differences using a Pearson’s chi squared test.

#### 2.4.2 Spatiotemporal overlap

We calculated the daily temporal patterns of activity, by season for images classified as coyotes, humans (without dogs), and dogs (with or without their owners). Overlap in activity (Δ_4_ and a 95% CI from bootstraps; Meredith & Ridout, 2014; Rowcliffe, 2021) was estimated for each species pair in each season. Analysis was conducted using the packages ‘*overlap’* and ‘*activity*’ in the program R (R Core Team, 2021).

The spatial analysis consisted of modelling the intensity of use (events/day) for each camera location in each season for images classified as coyotes, humans, and dogs using a general linear mixed modelling approach (Fox, 2016). For each group (coyotes, humans, and dogs) we developed a single model based on a similar set of covariates: season (spring as the reference category), whether the camera site was on a maintained trail, the distance (m) of the camera to the nearest vehicle parking area, and the intensity of use of the other two species at each site (i.e., intensity of use for humans and dogs were included in the coyote model). Only covariates with a Pearson’s correlation coefficient | r | < 0.6 were included in each model to avoid collinearity. We also included camera site as a random intercept to account for their repeated sampling. Models were fit using the ‘glmmTMB’ package framework and were evaluated using Nagelkerke’s r2 using the ‘performance’ package in the program R (Brooks et al., 2017; Lüdecke et al., 2021; R Core Team, 2021).

## 3 Results

Out of 137 wild canid fecal samples collected during 2018 in the BPRA, 18 were positive for *E. multilocularis*, leading to a true prevalence estimate of 15.7% (9.7-22.7%, 95% CrI). No significant seasonal variation was detected (chi-square=1.77, df=3, p=0.63; **Table 1**). The distribution of positive samples was relatively even throughout the park and did not represent any specific hotspots of infection (**Figure 1**).

**Table 1:** *Echinococcus multilocularis* prevalence in the BPRA estimated using qPCR of fecal samples by species and season.

Species	Season	Samples	Positive	Prevalence (%)	Lower CrI	Upper CrI
Coyotes	Spring	40	4	13.7	4.7	26.5
	Summer	40	6	19.2	8.2	33.7
	Fall	19	4	27.2	9.9	50.0
	Winter	38	4	14.2	4.9	27.5
Dogs	Summer	44	0	2.4	0.0	7.2

True prevalence was calculated using a test sensitivity of 87.1 and specificity of 100 along with 2.5% and 97.5% credible intervals (CrI) or 0% and 95% CrI (for samples with zero positive cases). Overall coyote prevalence was estimated as 15.7% (9.7-22.7%) with no statistical difference between seasonal prevalence (chi=1.77, df=3, p=0.63).

We collected a total of 44 owner surveys and related fecal samples from dogs that were frequently walked during the summer in the BPRA. No samples were positive for *E. multilocularis* infection following molecular analysis. Based on the test specificity and sensitivity this may still indicate that, if the parasite is circulating in this cohort of dogs, the prevalence could be of 2.4% (0-7.2, 95% CrI; **Table 1**). Survey results indicated the average age of dogs walked in the BPRA was 5.9 years (± 4.0 SD) with approximately equal representation of males (54.5%) and females (45.5%). A vast majority of the dogs were either spayed or neutered (93.2%) with only 40.9% being purebred. Owners reported that 90.9% of dogs had been to a veterinarian clinic in the last year and 72.7% had been dewormed in the same period (i.e. a factor potentially contributing to lower parasite prevalence). Within the BPRA, 36.4% of owners reported walking their dog off leash at least some of the time, 65.9% of dogs were reported to chase rodents, and 81.8% of dogs ate objects found on the ground, which included 45.5% of owners who reported that their dog sometimes to always ate found feces (i.e. potential parasitism risk factors). Additionally, 18.1% of owners reported feeding their dog hunting scraps.

**Figure 2:**
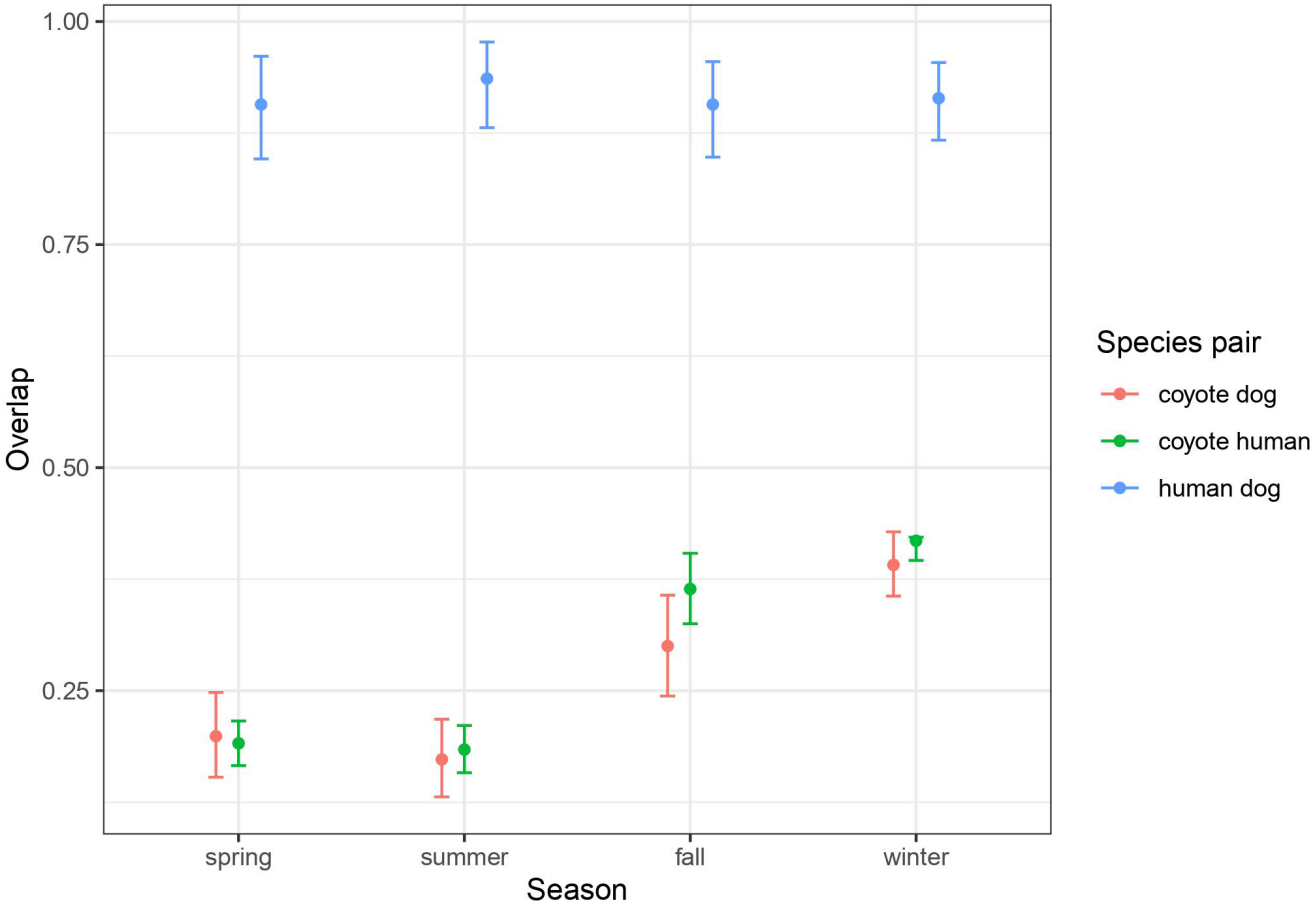
Average (and 95% CI) of daily activity overlap between species pairs by season, calculated from 37 remote cameras deployed from June 2017 to July 2018 throughout the study area. The daily patterns in activity for each season and species pair is given in the **supplemental material**, where the overlap is shown.

Using remote cameras distributed throughout the BPRA (**Figure 1**) we recorded 3459 coyote events, 8108 human events, and 631 dog events. Dogs were usually recorded alongside humans, however in 8.1% of dog events no humans were present. Using these events, we were able to estimate the seasonal overlap in temporal activity of coyotes, humans, and dogs. We found that humans and dogs were diurnally active and generally within the posted operating hours of the reserve (07:00-23:00). Humans and dogs naturally had very high levels of overlap (>0.9) across all seasons (**Figure 2**). Coyote activity exhibited a nocturnal pattern in spring, but gradually shifted to a cathemeral pattern in winter (daily temporal overlap plots for each season are given in the supplemental information). Based on inspection of the confidence intervals, this pattern of increasing mid-day activity results in an increase in overlap with humans and dogs in the fall and winter (0.300-0.418) seasons relative to spring and summer (0.173-0.199; **Figure 2**).

**Figure 3:**
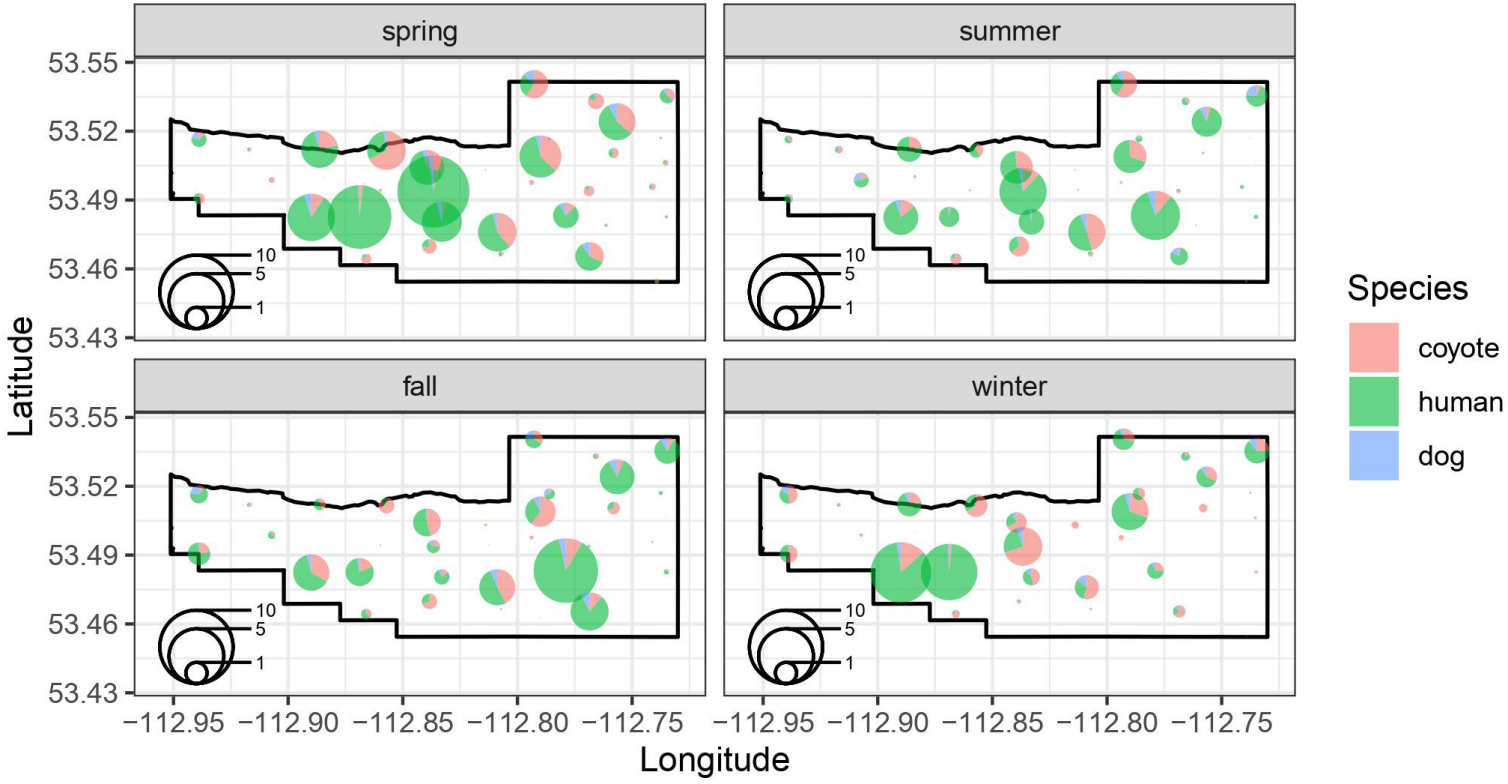
Seasonal spatial patterns in the locations of species-specific intensity of use (events/day) at each of the 37 remote camera location in the BPRA. The size of the pie chart at each location reflects the intensity of use (events per day) at a site, whereas colors represent the proportion of the events at a site that were coyotes, humans, and dogs.

We estimated the seasonal intensity of use (events/day) for coyotes, humans, and dogs based on a group of shared covariates related to the location of the camera and the attraction or avoidance of the other species intensity of use at a site (**Figure 3**). For coyotes, our model suggests that intensity of use is reduced in summer (p=0.03) and fall (p=0.04) relative to spring, but not in winter (p=0.71) (**Table 2**). Coyote intensity of use was higher at camera sites located on a maintained trail (p=0.03) and we detected a tendency to increase further away from vehicle parking areas (p=0.09). They also had greater intensity of use at sites where dog intensity of use was higher (p<0.001) but reduced, though not significantly, where human intensity of use was higher (p=0.13; **Table 2**). The model fit coyote intensity of use data well (conditional r^2 =^ 0.56), with the marginal component explaining approximately half of the explained variance (r^2 =^ 0.25, **Table 2**).

**Table 2:** Model coefficients and p-value for the random effect model of seasonal intensity of use (events/day) for coyotes, humans, and dogs.

Variables	Coyotes		Humans		Dogs	
	B	p	B	p	B	p
Intercept	-0.0212	0.85	-0.6432	0.09	0.0538	**<0.01**
Summer	-0.1041	**0.03**	-0.2457	0.25	-0.0006	0.94
Fall	-0.0993	**0.04**	-0.3425	0.11	0.0071	0.38
Winter	-0.0177	0.71	-0.2128	0.32	-0.0084	0.29
On trail	0.1661	**0.03**	0.4348	0.10	0.0127	0.35
Dist. to parking	0.00005	0.09	0.00024	**0.01**	-0.00001	**<0.01**
Coyotes	–		-0.4341	0.20	0.0562	**<0.01**
Humans	-0.0297	0.13	–		0.0187	**<0.01**
Dogs	2.017	**<0.01**	0.1202	**<0.01**	–	
Site	0.029 (0.173)		0.241 (0.491)		0.001 (0.031)	
r^2^	0.56 (0.25)		0.51 (0.37)		0.69 (0.43)	

Seasons are categorical variables and spring is the reference season, trail (0/1) is whether the camera was on a maintained trail, distance to nearest visitor parking (in meters), and the intensity of use of the other species (events/day) at a camera. The variance (std. dev) of the random effect of camera site is also given along with the conditional (and marginal) r^2^. Values in bold indicate the variables that were significant in the species-specific model, where a variable was not included in one of the models a dash (-) indicates the variables absence.

Human intensity of use did not differ by season (all p>0.11), was marginally higher on trails (p=0.10) and was further from vehicle parking areas (p=0.01). Human intensity of use was higher at sites where dog intensity of use was higher (p<0.001) and unrelated to coyote density of use (p=0.20; **Table 2**). The model fit the human intensity of use data reasonably well (conditional r^2 =^ 0.51) with the marginal component explaining most of the explained variance (r^2 =^ 0.37, **Table 2**).

Our model suggested that dog intensity of use was not related to season (all p>0.29), and not significantly higher at camera sites on maintained trails (p=0.35). However, intensity of use was significantly related to distance to vehicle parking areas (p=0.003) where sites closer to parking areas had higher intensity of use. Likewise, dog intensity of use was significantly higher where both human (p<0.001) and coyote (p<0.001) intensity of use was higher (**Table 2**). The dog model fit the intensity of use data very well (conditional r^2 =^ 0.69) with the marginal component explaining approximately two thirds of the explained variance (r^2 =^ 0.43, **Table 2**).

## 4 Discussion

If the risk of zoonotic transmission is the conditional probability of abundance and opportunity, then local prevalence needs to be understood within the context of host spatial and temporal distributions, and models of human use and risk factors that combine prevalence and spatiotemporal distribution (Deplazes et al., 2004; Atkinson et al., 2013; Liccioli et al., 2015). In this paper we have shown how this might be the case in a multiuse recreation area close to a known, emerging hotspot of zoonotic infections in humans in North America.

Our estimated *E. multilocularis* prevalence in coyotes (15.7%) was similar to, or higher than, other Canadian rural samples (0-24%; Gesy et al., 2014; Schurer et al., 2018; Kotwa et al., 2019; Kotwa et al., 2020; Sugden et al., 2020) but lower than other rural samples (35-72%; Gesy et al., 2013; Gesy et al., 2014; Kolapo et al., 2021; Steckler, 2021). Relative to more local urban samples, our reported prevalence is approximately half of that found in urban coyotes 40km west in nearby Edmonton, Alberta where prevalence of up to 80% has been reported (Catalano et al., 2012; Luong et al., 2020; Steckler, 2021), but is more similar to that of urban coyotes found 300km south in Calgary, Alberta (21.4%; Liccoli et al., 2014). While methodological differences may make direct comparisons difficult our approach estimating true prevalence based on our specific test sensitivity and specificity will allow unbiased comparisons in the future (Toews et al., 2021).

Coyotes are not the only wild definitive host for E. multilocularis in this region, although they may be the most likely to be involved in local transmission from the sylvatic to the synanthropic lifecycles. In their review of North American studies on E. multilocularis, Massolo et al. (2014) reported relatively few studies on red foxes relative to coyotes, despite a similar prevalence, a result confirmed in Ontario (Kotwa et al., 2019). This is very different from the situation in Europe where red foxes are the most important definitive hosts and route of transmission to domestic dogs and humans (Oksanen et al., 2016). It is very likely that this is the result of mesocarnivore release, whereby the removal of wolves in the North American system has allowed coyote number to rebound and subsequently resulted in a decline in red foxes (Crooks and Soule, 1999, Gosselink et al., 2007; Levi and Wilmers, 2012). Our single fox event among 3459 coyote events seems to bear this trophic consequence out in our study area. This also seems to be the case in the nearby city of Edmonton where a recent city-wide remote camera study found that coyotes have become much more common (7662 detections) relative to red foxes (21 detections; Stevenson, 2022) further highlighting the potential importance of coyotes in the *E. multilocularis* life cycle across a gradient of urbanization in western North America.

While we found no positive cases of *E. multilocularis* within the dog samples analyzed here, however, after accounting for the sensitivity and specificity of the test we estimated that, if present in dogs, the parasite could be at a true prevalence of 2.4%. We note that our sample size (n=44) was relatively low, yet it is in line with recent studies in urban centres in Alberta have reported a much lower (Edmonton=0.2%, Porter et al., 2022) or similar infection rate (Calgary=2.4%, Toews, 2020), both with considerably larger sample sizes. Further research is needed to estimate the prevalence for Alberta’s dog population, particularly those outside of urban centers, which a recent global review of *E. multilocularis* has indicated may increase the risk of infection in humans (Toews et al., 2021).

In contrast to Liccoli et al. (2014) that reported spring infection rates roughly twice that of the other seasons for urban coyotes, we found no significant seasonal trend in coyote infections. Seasonal differences may be the result of different diets, particularly the seasonal consumption of rodents which are intermediate hosts for *E. multilocularis* (Liccioli et al., 2014; Liccioli et al., 2015). Pruss (2002), in the adjacent Elk Island National Park (Alberta), noted that rodents (e.g., mice, voles, lemmings, and muskrats) occurred in 70% of coyote scats and comprised the dominant food type in 53% of scats with a seasonal peak in fall, corresponding to when we observed the highest infections in coyotes. However, infection may not only rely on the relative abundance of intermediate hosts in coyote diets. Mori et al. (2019) suggested that the relative composition of the prey assemblage in terms of proportion of competent prey species in the diet, and the epidemiology of the infections in the intermediate hosts (i.e., stage of infection, and relative abundance of infectious hosts) may play even a more relevant role than diet itself. Infection (host competency) in definitive hosts typically occurs one month following ingestion of infected rodents and the host may shed eggs for the following two months, approximately (Kapel et al., 2006). While reinfection is possible, and total immunity is unlikely, worm burden has been experimentally been shown to be greatly reduced (Torgenson, 2006, Kouguchi et al., 2016). Thus, a more intense sampling of infections in both definitive and intermediate hosts, along with an analysis of the diet of the definitive hosts, may help understanding these processes at local scales.

Our models using remote camera observations of coyotes, dogs, and humans suggested that coyote intensity of use was higher on maintained trails where humans’ intensity of use for recreation was highest and, although coyotes use was higher further from vehicle parking areas, they had a greater intensity of use where dog use was higher, indicating a potential for interactions. Seasonal patterns of temporal activity suggested that coyotes and dogs activity, and humans overlap the most during the fall and winter seasons, likely induced by the shortened daylight hours. Fall and winter seasons, in which the activity of human/dogs and coyotes had the highest overlap in this study, also present conditions under which *E. multilocularis* eggs may retain their viability for the longest amount of time, potentially resulting in an increased environmental load (Veit et al., 1995). This buildup of shed eggs in the environment *via* coyotes may result in increased local exposure for humans, either through secondary transfer of the eggs from scavenging dogs to humans or potentially increased prevalence of infectious rodents that may be preyed by dogs resulting in intestinal infections and consequent shedding of eggs (Torgenson and Heath, 2003). Indeed, a majority (65.9%) of dog owners in the BPRA reported that their dogs chased rodents. It is still unknown which of these pathways may be the most problematic for human cases, although amongst AE cases in Alberta, 88% of patients were dog owner, which appeared to the biggest shared risk factor (Houston et al., 2021).

Our results can inform the management of zoonotic risk (Deplazes et al., 2004). Veterinary care including the regular treatment of dogs with anthelminthic drugs (i.e., praziquantel) is a pivotal step in the management of infection in domestic dogs (Deplazes et al., 2011; Kotwa et al., 2019). Management of *E. multilocularis* aiming at wild definitive hosts *via* baiting with anthelminthic drugs when the likelihood of transmission is higher (e.g. fall and winter as for this study) would be an alternative strategy, but requires significant planning and effort that may infeasible across a large natural area like the BPRA (Hegglin et al., 2003; Hegglin and Deplazes, 2008; Hegglin and Deplazes, 2013; Umhang et al., 2019). Indeed, preventative actions aimed at the dogs of those who recreate in the BPRA and public education about the risks associated may be more suitable for limiting the infection potential of *E. multilocularis* (Deplazes et al., 2011). Our results advocate for taking a more integrated approach focusing on preventative actions to reduce risk for infections at three levels: through regular protocols for deworming dogs that are walked in areas where the parasite is endemic (>0.10 of prevalence in the sylvatic definitive hosts); by increased communication and information strategies targeting park users to implement more stringent procedures for sanitizing berries in these areas, as well as paying particular attention to hand-washing and general hygiene to reduce the risk of hand-mouth transmission; and finally, in those areas where the activity overlap is critical, and the prevalence in wild definitive host is particularly high (>0.50), also baiting with anthelminthic drugs should be considered, but with very site-specific predetermined protocols with ex-ante and ex-post assessments. This integrated approach, typical of both One-health and Eco-health paradigms, besides being efficient, also helps to better understand the potential zoonotic risks and inform managers to minimize the risks to public health at the interface of wildlife, domestic animals and people (Webster et al., 2016; Lerner and Berg, 2017).

## Data availability statement

The raw data supporting the conclusions of this article will be made available by the authors, without undue reservation.

## Ethics statement

The studies involving human participants were reviewed and approved by The King’s University Research Ethics Board. The patients/participants provided their written informed consent to participate in this study. The animal study was reviewed and approved by The King’s University Research Ethics Board. Written informed consent was obtained from the owners for the participation of their animals in this study.

## Author contributions

DV, MM, and AM contributed to conception and design of the study. ET, JP, PW, and CK helped collect the data. ET analyzed the biological samples and DV performed the statistical analysis. ET, JP, PW, and CK wrote sections of initial drafts. DV wrote the first draft of the manuscript. All authors contributed to the article and approved the submitted version.
