# Supplementary material for: Host spatiotemporal overlap in a park with high endemicity of Echinococcus multilocularis
Source: Front Parasitol. 2023 Mar 13;2:1161108. doi: 10.3389/fpara.2023.1161108 (PMC11732005; doi:10.3389/fpara.2023.1161108)
Supplement: Supplementary file 1 [file DataSheet_1.docx]

Supplementary Material

Host spatiotemporal overlap in a park with high endemicity of Echinococcus multilocularis

**Darcy R. Visscher*, Emilie Toews, Jesse Pattison, Philip D. Walker, Colborne Kemna, Marco Musiani, and Alessandro Massolo**

*** Correspondence:** Corresponding Author: darcy.visscher@kingsu.ca

# Supplementary Figures and Tables

## Supplementary Figures

**Supplementary Figure 1.** Daily temporal activity for spring, summer, fall, and winter (given as rows, top to bottom, respectively) and overlap (grey shaded) for species interactions where coyotes (red) and humans (black) are compared in the left column, coyotes (red) and dogs (blue) are compared in the middle column, and humans (black) and dogs (blue) are compared in the right column. The 24-hour clock time is given on the x-axes and the density of events are given on the y-axes.

# Supplementary Figures and Tables

## Dog Owner Questionnaire and Survey Tool

***Screening Questions:***

1. Are you over the age of 18? **Yes No**

2. Is this your dog(s)? **Yes No**

3. Does your dog normally defecate in this park? **Yes No**

***General:***

4. What is your dog’s name? **__________________**

5. What is the age of your dog? **_____**

6. What is the breed of your dog? **Purebred: __________ Cross: _____________ Mixed**

7. What is the sex of your dog? **Male Female**

8. Is your dog neutered/spayed? **Yes No**

9. Has your dog visited a vet in the last year? **Yes No**

10. Has your dog been dewormed in the last year (including heartworm)? **Yes No**

***Dog Walking Activities:***

11. How often, if at all, have you spent time with your dog in the following areas in the last **4 months:**

| **Blackfoot/Cooking Lake Area:** | Never | Less than 1x/month | 1x/month | 1x/month to 3x/month | 1x/week to 6x/week | Daily |
| --- | --- | --- | --- | --- | --- | --- |
| **City Dog Parks:** | Never | Less than 1x/month | 1x/month | 1x/month to 3x/month | 1x/week to 6x/week | Daily |
| **Sidewalk/Street:** | Never | Less than 1x/month | 1x/month | 1x/month to 3x/month | 1x/week to 6x/week | Daily |
| **Sport/School Field:** | Never | Less than 1x/month | 1x/month | 1x/month to 3x/month | 1x/week to 6x/week | Daily |
| **Provincial/National Parks (**excluding Elk Island**):** | Never | Less than 1x/month | 1x/month | 1x/month to 3x/month | 1x/week to 6x/week | Daily |
| **Farm/Ranch/Acreage:** | Never | Less than 1x/month | 1x/month | 1x/month to 3x/month | 1x/week to 6x/week | Daily |

12. How often, if at all, do you walk your dog **off-leash** in the following areas:

| **Blackfoot/Cooking Lake Area:** | Never | Rarely | Sometimes | Often | Most of the Time | Always |
| --- | --- | --- | --- | --- | --- | --- |
| **City Dog Parks:** | Never | Rarely | Sometimes | Often | Most of the Time | Always |
| **Sidewalk/Street:** | Never | Rarely | Sometimes | Often | Most of the Time | Always |
| **Sport/School Field:** | Never | Rarely | Sometimes | Often | Most of the Time | Always |
| **Provincial/National Parks (**excluding Elk Island**):** | Never | Rarely | Sometimes | Often | Most of the Time | Always |
| **Farm/Ranch/Acreage:** | Never | Rarely | Sometimes | Often | Most of the Time | Always |

***Dog Feeding Behaviour***

13. Does your dog chase rodents while on walks? **Yes No**

**If yes,** how often is your dog successful in catching rodent prey on walks?

**Never Rarely Sometimes Often Most of the Time Always**

14. Does your dog eat things it finds on the ground while on walks? **Yes No**

**If yes,** how often does your dog eat **feces** it finds while on walks?

**Never Rarely Sometimes Often Most of the Time Always**

**If yes,** how often does your dog eat **rodent/other animal carcasses** it finds while on walks? **Never Rarely Sometimes Often Most of the Time Always**

15. At home, what do you primarily feed your dog?

**Dry Kibble Wet/Canned Food Homemade (veggie based) Homemade (meat based)**

16. Has your dog ever been fed offal from hunted deer/elk/moose? **Yes No**

**If yes**, how many times **in the last year**? **___**

***Contact Information:***

Name of dog owner: **_____________________**

Phone number of dog owner: **(_____)-_____-________**

E-mail address of dog owner: **____________________**

Preferred method of contact: **____________________**
